# Opposite effects of spermidine and GC7 in cell culture are dictated by distinct molecular targets

**DOI:** 10.1042/BCJ20253298

**Published:** 2025-12-17

**Authors:** Tomoaki Tahara, Rosa Bordone, Antonio Francesco Campese, Noemi Martina Cantale Aeo, Roberta Astolfi, Sonia Coni, Rino Ragno, Gianluca Canettieri, Enzo Agostinelli

**Affiliations:** 1 Department of Molecular Medicine, Sapienza University of Rome, Rome, 00161, Italy; 2 Department of Sensory Organs, Sapienza University of Rome, Rome, 00161, Italy; 3 Rome Center for Molecular Design, Department of Drug Chemistry and Technology, Sapienza University, Rome, 00185, Italy; 4 International Polyamines Foundation ONLUS-ETS, Rome, 00159, Italy

**Keywords:** bovine serum amine oxidase, BSAO, deoxyhypusine synthase, DHPS, eIF5A, N1-guanyl-1,7-diaminoheptane, GC7, spermidine, SPD, molecular docking

## Abstract

Spermidine (SPD) and related polyamines are small polycationic molecules typically elevated in cancer cells, where their depletion suppresses tumor growth both *in vitro* and *in vivo*. Paradoxically, SPD has also been proposed as a dietary supplement for its potential health benefits, including cancer prevention, prompting considerable interest in elucidating its mechanisms of action. *In vitro* studies using cultured cancer cell lines treated with exogenous SPD have yielded conflicting results, with reports of enhanced proliferation, cytotoxicity, or modulation of autophagy. To address these discrepancies, we used polyamine-depleted colorectal cancer (CRC) cells to systematically evaluate SPD's effects across a range of concentrations. Following depletion with difluoromethylornithine, SPD exhibited a biphasic response: at low concentrations (<20 µM), it promoted proliferation via deoxyhypusine synthase (DHPS)-dependent hypusination of eukaryotic initiation factor 5A, whereas high concentrations (>100 µM) induced DHPS-independent cytotoxicity mediated by bovine serum amine oxidase (BSAO) activity in fetal bovine serum. High SPD doses transiently inhibited the autophagic flux, while low doses did not display any effect at all time points tested. The DHPS inhibitor GC7 (N^1^-guanyl-1,7-diaminoheptane) suppressed SPD-induced proliferation at low concentrations and unexpectedly prevented cytotoxicity at high concentrations. Kinetic assays revealed that GC7 also inhibits BSAO in a non-competitive manner (Ki ≈ 300 nM), independent of DHPS. *In silico* docking analysis indicated that GC7 binds BSAO via non-covalent interactions, outside the topaquinone organic cofactor site. These findings clarify the concentration-dependent effects of SPD in CRC cells, reconcile conflicting *in vitro* data, and identify BSAO as a previously unrecognized target of GC7, providing new mechanistic insights into polyamine-driven cancer biology.

## Introduction

Polyamines are small polycationic molecules derived from arginine that play essential roles in cellular homeostasis. The polyamine biosynthetic pathway is initiated by ornithine decarboxylase (ODC), a MYC-regulated enzyme [1] that catalyzes the rate-limiting decarboxylation of ornithine to produce putrescine. Putrescine is subsequently converted to spermidine (SPD) and spermine through the sequential addition of 3-aminopropyl groups. These polyamines regulate critical cellular processes, including proliferation, metabolism, transcription, translation, and polyamine homeostasis [2,3].

One key substrate of SPD is eukaryotic initiation factor 5A (eIF5A), which undergoes a unique covalent modification known as hypusination. This process involves the transfer of a SPD moiety to eIF5A by deoxyhypusine synthase (DHPS), followed by hydroxylation by deoxyhypusine hydroxylase (DOHH), enabling eIF5A to perform its essential translational functions [4].

Polyamine metabolism and hypusination are frequently up-regulated in cancer cells, where they support rapid proliferation, invasion, and metastasis. Additionally, emerging observations highlight the role of polyamines in regulating various cell types of the tumor microenvironment, including immune cells, fibroblasts, and endothelial cells to support cancer growth [5]. Of note, high polyamine levels sustain growth and function of immunosuppressive cell types such as Myeloid-Derived Suppressor Cells (MDSCs), macrophages, and regulatory T-cells, while polyamine depletion could bust tumor response to immunotherapy, motivating efforts to therapeutically target these pathways.

The irreversible ODC inhibitor difluoromethylornithine (DFMO) has shown efficacy as a cytostatic agent, particularly in cancer prevention trials. Promising results have been observed in patients with familial adenomatous polyposis (FAP) and in high-risk populations for colorectal cancer (CRC) [6]. However, chronic DFMO administration often leads to compensatory up-regulation of polyamine transporters, allowing cancer cells to replenish intracellular polyamine pools from extracellular sources.

To overcome this limitation, downstream effectors of polyamines, such as eIF5A, have been explored as alternative therapeutic targets. Recent studies revealed that hypusinated eIF5A promotes MYC translation and CRC growth. Inhibition of DHPS with N1-guanyl-1,7-diaminoheptane (GC7), a SPD analog, suppresses hypusination, resulting in CRC growth inhibition and reducing MYC expression *in vitro* and in APC^min^ mice [7]. The combination of GC7 with DFMO enhances these effects, demonstrating synergistic inhibition and cytotoxicity of CRC growth [8]. However, previous publications have revealed that GC7 may act independently of eIF5A, although the mechanisms of these presumed off-target effect have not been characterized [9,10].

Interestingly, while reducing SPD levels or inhibiting DHPS activity has shown efficacy in preventing cancer growth [7,8], other observations provided evidence that SPD treatment induces toxicity in cell culture, owing to the presence in the ruminant serum of bovine serum amine oxidase (BSAO) [11,12], a copper enzyme that deaminates polyamines to generate the cytotoxic products H_2_O_2_ and the aldehyde acrolein.

Since cell culture models have been often used to elucidate the mechanism of action underlying the biological and functional response to SPD supplementation or to inhibitors of SPD targets, in this study, we have addressed these conflicting results using polyamine-depleted cancer cell cultures reconstituted with varying concentrations of SPD. We observed a dose-dependent, dual effect of SPD, with low doses promoting proliferation via eIF5A hypusination and MYC translation, while high doses induced cytotoxicity via BSAO. Notably, we discovered a novel target of GC7 by demonstrating that this compound not only inhibits DHPS but also binds BSAO and inhibits its activity, abrogating the oxidative toxicity of high SPD doses.

## Results

### Extracellular SPD induces a dual effect on polyamine-depleted CRC cells

**Figure 1:**
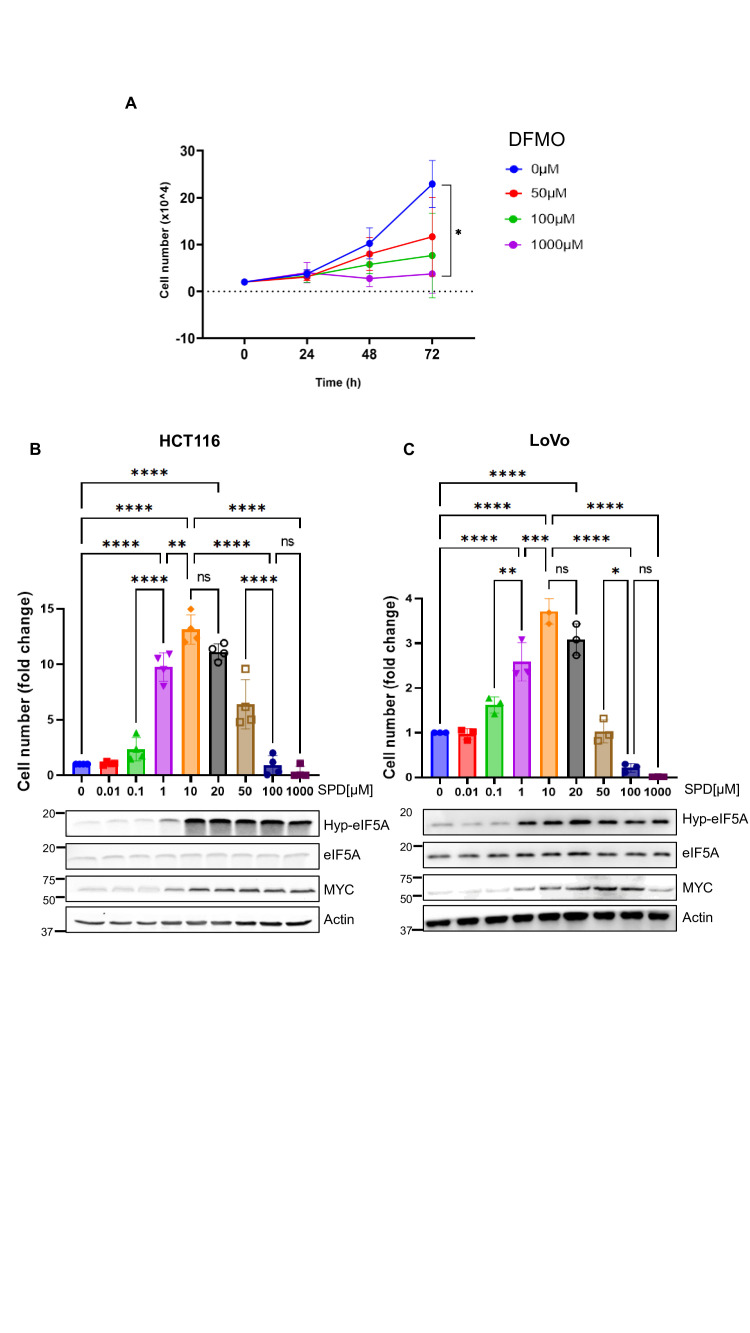
Dual effect of SPD in polyamine-depleted colon cancer cells. (**A**) Cell proliferation assay of HCT116 cells (*n* = 3) treated with different concentrations of DFMO (0, 50, 100, and 1000 µM) for 24, 48, and 72 hours. Data represent the mean ± SD of experiments performed in triplicate. For statistical analysis, *P<0.05 was determined by Tukey’s multiple comparisons test following one-way ANOVA. (**B**) Upper panel: cell proliferation assay in HCT116 cells (*n* = 3) pre-treated with 1 mM DFMO for 72 hours then exposed to the indicated concentrations of SPD for 72 hours. Data represent the mean ± SD of experiments performed in triplicate. For statistical analysis, **P<0.01, ****P<0.0001, were determined using Dunnett’s multiple comparisons test following one-way ANOVA. Bottom panel: Western blot analysis showing hypusinated eIF5A (Hyp-eIF5A), eIF5A, MYC, and actin (loading control) in HCT116 cells pre-treated with 1 mM DFMO and the indicated concentrations of SPD for 48 hours. (**C**) Upper panel: cell proliferation assay in LoVo cells (*n* = 3) pre-treated with 1 mM DFMO for 72 hours then exposed to the indicated concentrations of SPD for 72 hours. Data represents the mean ± SD of experiments performed in triplicate. For statistical analysis, ns = not significant (*P*>0.05), *P<0.05, **P<0.01, ***P<0.001, ****P<0.0001, were determined using Dunnett’s multiple comparisons test following one-way ANOVA. Bottom panel: Western blot analysis showing hypusinated eIF5A (Hyp-eIF5A), eIF5A, MYC, and Actin (loading control) in LoVo cells pre-treated with 1 mM DFMO and the indicated concentrations of SPD for 48 hours.

Given the reported contradictory effect of SPD supplementation in colon cancer [13–16], we sought to carry out a detailed dose-response analysis in HCT116 cells, a widely used human CRC cell line that is easy to maintain, highly transfectable, amenable to genetic manipulation, and previously shown to respond to polyamine modulation [7,8]. To deplete the cells from the endogenous polyamine pool, we treated them with DFMO, an irreversible ODC inhibitor. As expected, DFMO inhibited cell growth in a dose-dependent manner, with the strongest effect achieved at 1 mM concentration, which caused a complete inhibition of cell proliferation (Figure 1A). Hence, we used this dose for 72 hours to achieve intracellular polyamine depletion.

DFMO-treated cells were incubated with a wide range of SPD concentrations (10 nM–1 mM) for 72 hours, and cell number was measured after an additional 72 hours. As shown in Figure 1B, SPD concentrations within the low micromolar range significantly increased cell proliferation in a dose-dependent manner, reaching a plateau at 10 µM. When the concentration of SPD reached 50 µM, the number of cells started being significantly reduced compared with the previous doses, and at 100 µM the proliferative effect was completely abrogated. We confirmed the observed dose-dependent effect of SPD on cell proliferation also in Lovo cells, another widely used polyamine-dependent human CRC cell line, ruling out the involvement of a cell-specific response (Figure 1C).

Based on our previous work showing that SPD supplementation promotes eIF5A-mediated MYC translation [7], we tested the role of this mechanism in the observed dual effect.

Accompanying the dose-dependent increase in cell proliferation, eIF5A hypusination and total MYC levels were up-regulated starting from 1 µM SPD and reached a plateau at 10 µM (Figure 1B and C). At higher doses, hyp-eIF5A and MYC levels remained elevated, suggesting that this mechanism causes the initial proliferative response but not the subsequent decline of cell number observed at the higher doses of SPD.

**Figure 2:**
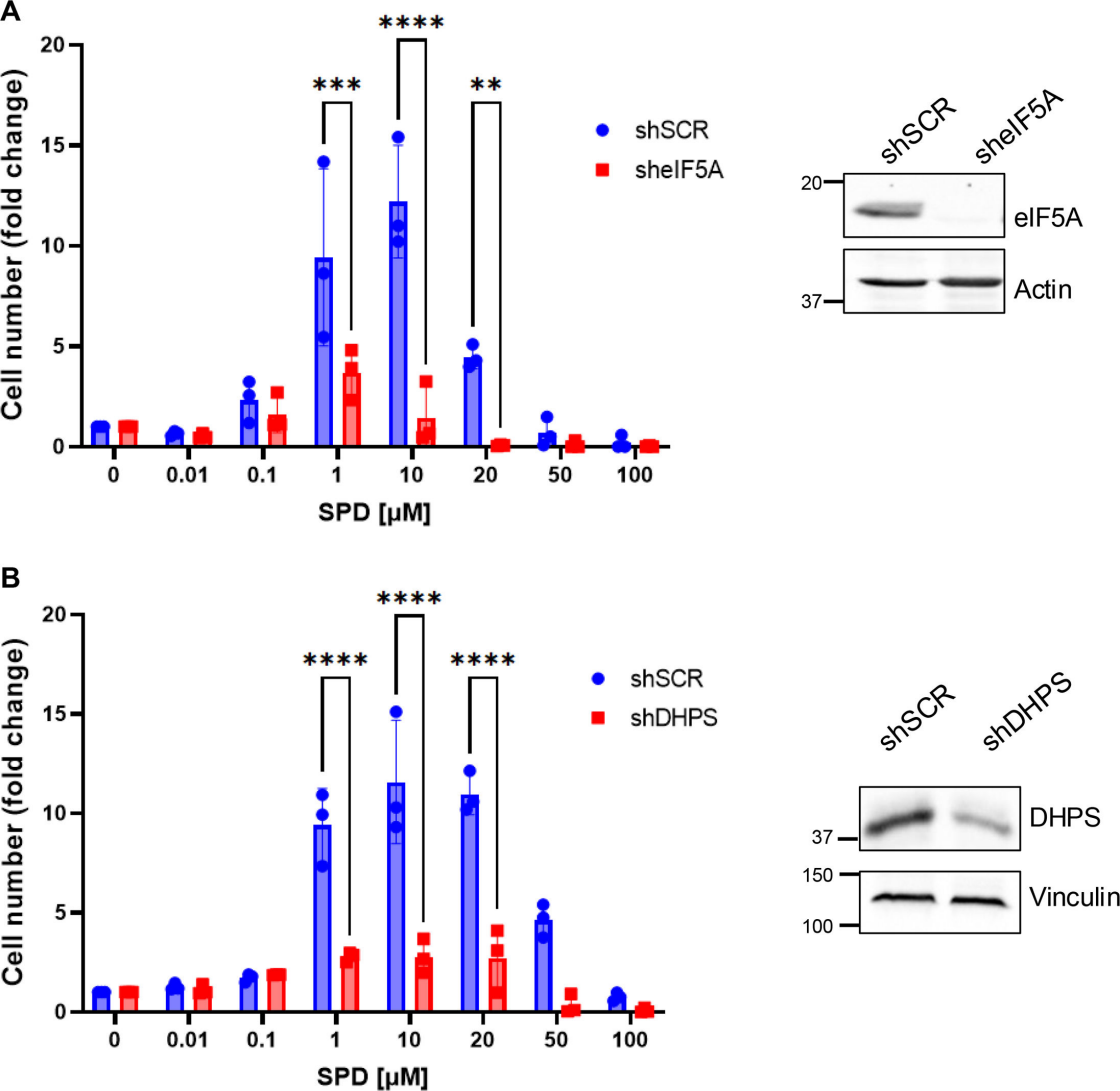
SPD induction of cell proliferation requires DHPS and eIF5A. (**A**) Left panel: proliferation assay of HCT116 cells (*n* = 3) transduced with a lentivirus expressing shRNA targeting eIF5A (sheIF5A) or control (shSCR), pre-treated with 1 mM DFMO for 72 hours, then treated with the indicated concentrations of SPD for 48 hours. Data represent the mean ± SD of experiments performed in triplicate. For statistical analysis, **P<0.01, ***P<0.001, and ****P<0.0001 were determined using Šidák’s multiple comparisons test following two-way ANOVA. Right panel: Western blot analysis showing eIF5A knock-down efficiency. (**B**) Left panel: proliferation assay of HCT116 cells (*n* = 3) transduced with a lentivirus expressing shRNA targeting DHPS (shDHPS) or control (shSCR), pre-treated with 1 mM DFMO for 72 hours, then treated with the indicated concentrations of SPD for 48 hours. Data represent the mean ± SD of experiments performed in triplicate. For statistical analysis, ****P<0.0001 was determined using Šidák’s multiple comparisons test following two-way ANOVA. Right panel: Western blot analysis showing DHPS knock-down efficiency.

To confirm this hypothesis, eIF5A or DHPS was ablated from cells using lentiviruses expressing gene-specific shRNAs. Knockdown of both genes significantly reduced the proliferative effect at the lower SPD doses (Figure 2A and B). Conversely, the SPD-induced inhibition of cell proliferation observed at the higher doses was not modified by depletion of DHPS or eIF5A, indicating that distinct targets and mechanisms regulate this effect. In keeping with this observation, administration of the polyamine transporter inhibitor AMXT-1510 prevented the proliferation advantage of low-dose SPD but failed in preventing the extracellular cytotoxic effect of the high-dose range (Supplementary Figure S4), supporting the hypothesis that the pro-proliferative effect is intracellularly mediated by eIF5A hypusination, while the inhibition of cell proliferation is linked to the extracellular content of SPD.

Moreover, SPD also inhibited cell viability in non-polyamine-depleted cells as indicated in Supplementary Figure S1, showing increased trypan blue positivity, although at lower doses, with an estimated EC50 of 18.23 µM. Under these conditions, the percentage of trypan blue positive cells was also dose-dependently increased (Supplementary Figure S1) in accordance with the reduction in cell number (Supplementary Figure S1), suggesting the occurrence of cell death.

### High doses of SPD promote apoptosis

**Figure 3:**
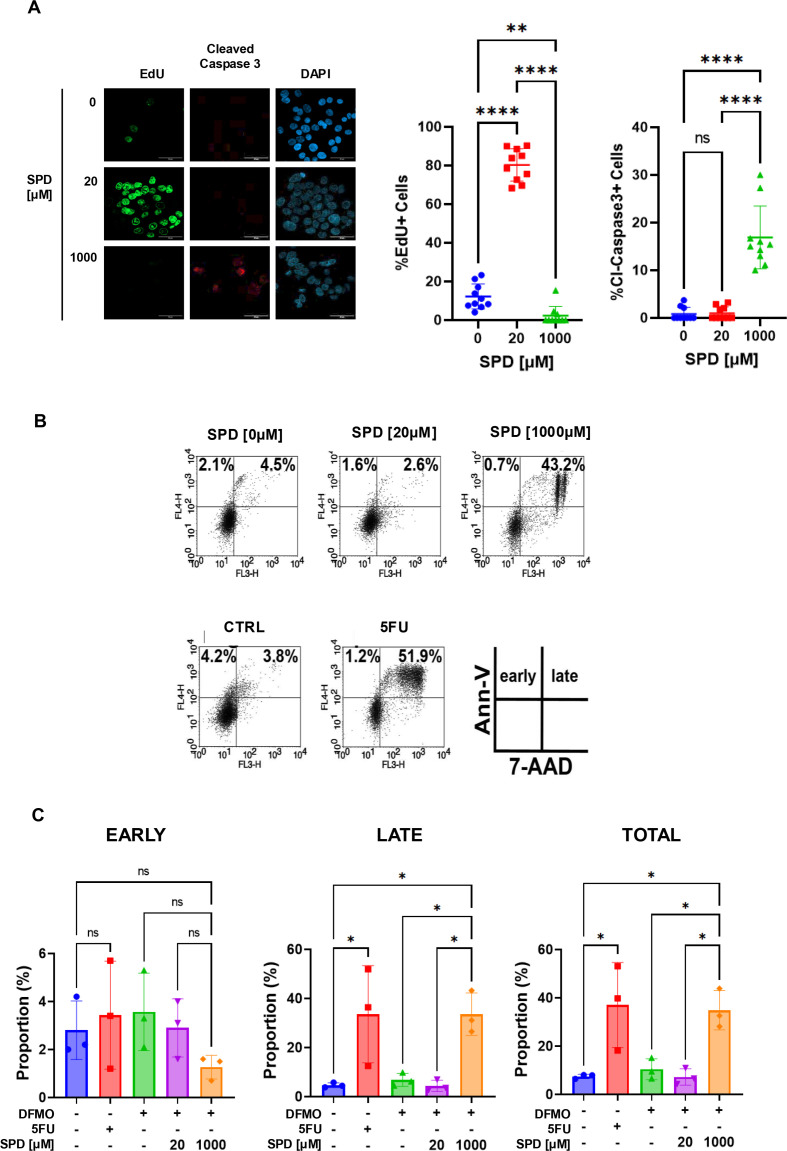
Effect of high and low doses of SPD on apoptosis. (**A**) Left panel: representative confocal images of immunofluorescence in HCT116 pre-treated with DFMO for 72 hours, then treated with 0, 20, or 1000 μM SPD for 72 hours. EdU was added to the media 6 hours prior to fixation, and cells were stained for DNA-incorporated EdU, Cleaved-Caspase 3 and DAPI for nuclear staining. Scale bar: 40 μm. Right panel: percentage of EdU and Cleaved-Caspase 3-positive cells related to the experimental setting described in A. For statistical analysis, ns = not significant (*P*>0.05), **P<0.01, ****P<0.0001 was determined using Tukey’s multiple comparisons test following one-way ANOVA. (**B**) Representative flow cytometry analysis showing the percentage of early and late apoptotic HCT116 cells stained with Annexin-V and 7-AAD. Cells were treated with 0, 20, or 1000 μM SPD after 72 h of pre-treatment with 1 mM DFMO. 50 μM 5FU was used as a positive control compared with vehicle-treated cells (CTRL). (**C**) Left panel: percentage of early apoptotic HCT116 cells treated as described in A (Annexin-V positive, 7-AAD negative cells); middle panel: percentage of late apoptotic cells (Annexin-V positive, 7-AAD positive cells); right panel: total apoptotic cells (early + late). Data represent the mean ± SD of three independent experiments. For statistical analysis, *P<0.05, ns = not significant (*P*>0.05) was determined using Tukey’s multiple comparisons test following one-way ANOVA.

To determine whether the reduction in cell number observed at high SPD concentrations was due to decreased proliferation and/or increased cell death, we administered the thymidine analog 5-ethynyl-2′-deoxyuridine (EdU), which is incorporated into newly synthesized DNA and detected by immunofluorescence, together with cleaved Caspase-3 to label apoptotic cells. We observed that low concentrations of SPD (20 μM) significantly induce cell proliferation without affecting cell death. Conversely, a high dose of SPD (1000 μM) abrogates cell proliferation and promotes Caspase-3 cleavage, documenting the involvement of both cell growth inhibition and apoptosis induction in the overall cytotoxic effect (Figure 3A). Supporting the observation that the antiproliferative effect observed at the higher doses was mediated by apoptosis, we performed flow cytometry analysis after Annexin V and 7-Aminoactinomycin D (7-AAD) staining of polyamine-depleted HCT116 cells. Cells were pre-treated with 1 mM DFMO to induce polyamine depletion and then were treated with SPD in the presence of DFMO for 48 hours. Compared with the untreated cells (6.6%), exposure to 20 µM SPD did not induce a significant change in the percentage of apoptotic cells. Conversely, after treatment with 1 mM SPD, there was a robust 6.6-fold increase in apoptotic cells (Figure 3B up - Figure 3C right) that was comparable to the response obtained with 50 µM 5Fu, used as a positive control (Figure 3B bottom – 3C right). Analysis of cell populations revealed that a significant fraction of the cells treated with high doses of SPD or 5Fu underwent late apoptosis, while the percentage of cells undergoing early apoptosis was not significantly changed by both treatments (Figure 3C middle, left panels).

### SPD administration impairs the autophagic flux

Since several reports suggest that SPD induces autophagy in human cells [17], likely through eIF5A-mediated translation of autophagic regulators [18], and since autophagy could be activated by oxidative stress, leading to apoptosis, we wondered whether the observed SPD-mediated effects could be attributed to variations in the autophagic flux.

**Figure 4:**
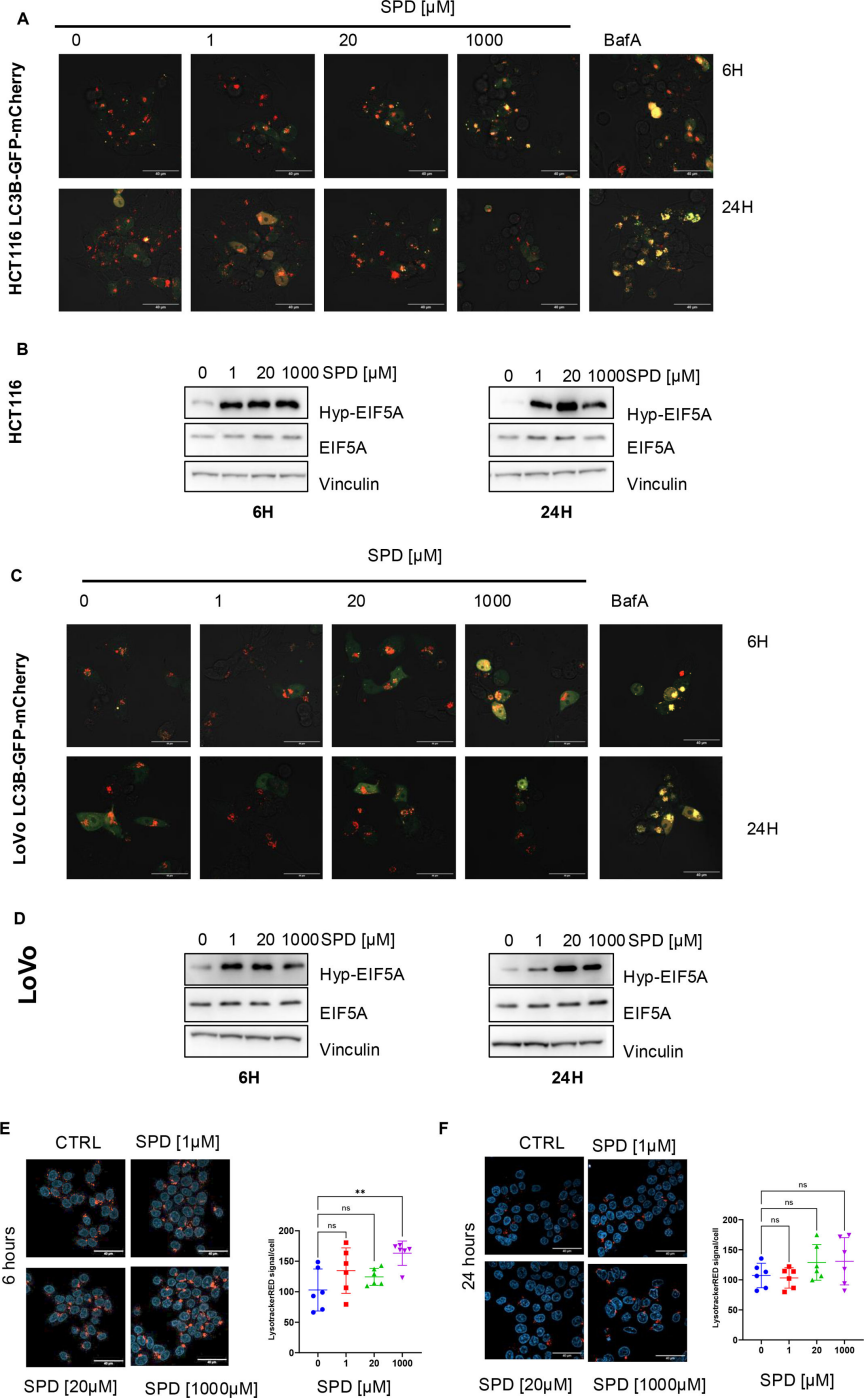
Effect of high and low doses of SPD on autophagy. (**A**) Representative confocal images of HCT116 cells transduced with the lentivirus expressing the mCherry-GFP-LC3, pre-treated with 1 mM DFMO for 72 hours, then treated with the indicated concentrations of SPD for 6 hours (top) or 24 hours (bottom). Yellow puncta indicate autophagosomes (mCherry+/GFP+), while autolysosomes appear red (mCherry+/GFP−). Scale bar 40 μm. (**B**) Western blot analysis showing hypusinated eIF5A (Hyp-eIF5A), total eIF5A, and Vinculin (loading control) in HCT116 cells pre-treated with 1 mM DFMO and the indicated concentrations of SPD for 6 hours (left) or 24 hours (right). (**C**) Representative confocal images of LoVo cells transduced with the lentivirus expressing the mCherry-GFP-LC3, pre-treated with 1 mM DFMO for 72 hours, then treated with the indicated concentrations of SPD for 6 hours (top) or 24 hours (bottom). Yellow puncta indicate autophagosomes (mCherry+/GFP+), while autolysosomes appear red (mCherry+/GFP−). Scale bar 40 μm. (**D**) Western blot analysis showing hypusinated eIF5A (Hyp-eIF5A), total eIF5A, and Vinculin (loading control) in LoVo cells pre-treated with 1 mM DFMO and the indicated concentrations of SPD for 6 hours (left) or 24 hours (right). (**E**) Left: representative confocal images of HCT116 cells treated for 6 hours with 1, 20, or 1000 μM SPD after 72 hours of pre-treatment with 1 mM DFMO. Before imaging, live cells were stained with Lysotracker Red (red puncta) and DAPI (blue). Scale bar 40 μm. Right: quantification of Lysotracker Red signal using ImageJ from 6 independent images normalized to the respective cell number. For statistical analysis, **P<0. was determined by Dunnett’s multiple comparisons test following one-way ANOVA. (**F**) Right: representative confocal images of HCT116 cells treated for 24 hours with 1, 20, or 1000 μM SPD after 72 hours of pre-treatment with 1 mM DFMO. Before imaging, live cells were stained with Lysotracker Red (red puncta) and DAPI (blue). Scale bar 40 μm. Right: quantification of Lysotracker Red signal using ImageJ from six independent images normalized to the respective cell number. For statistical analysis, ns = not significant (*P*>0.05) was determined by Dunnett’s multiple comparisons test following one-way ANOVA.

We assessed autophagic changes upon SPD administration in HCT116 and LoVo cells using the mCherry-GFP-LC3 reporter system [19]. We observed an increase in yellow puncta (autophagosomes) after 6 hours of treatment with high-dose SPD (1000 µM), similar to the autophagy inhibitor Bafilomycin A (Figure 4A and C), used as a control, suggesting a possible blockade in the autophagic flux, despite eIF5A hypusination (Figure 4B and D). Consistent with this hypothesis, the Lysotracker Red staining was significantly increased after 6 hours at the same SPD concentration (Figure 4E), supporting an impaired clearance of the autophagosome vesicles. Conversely, we did not observe any significant change in LC3B puncta formation (Figure 4A and C) or lysotracker staining (Figure 4E and F and Supplementary Figure S2) at any other concentration or time point despite induction of eIF5A hypusination (Figure 4B and D), indicating that only high doses of SPD induce a transitory, short-term inhibition of autophagy and arguing against the possibility of an eIF5A-mediated response.

### The cytotoxic effect of SPD requires its BSAO-mediated oxidation

Previous works demonstrated that SPD has a cytotoxic effect when cells are grown in medium supplemented with fetal bovine serum (FBS), as it contains BSAO, which catalyzes SPD oxidation with the release of the cytotoxic agents acrolein and hydrogen peroxide [20].

To verify that the cytotoxic effect observed with high SPD doses could be attributed to this mechanism, we first treated polyamine-depleted CRC cells with increasing amounts of SPD, alone or together with 500 µM aminoguanidine (AG), an amine oxidase (AO) inhibitor [21].

**Figure 5:**
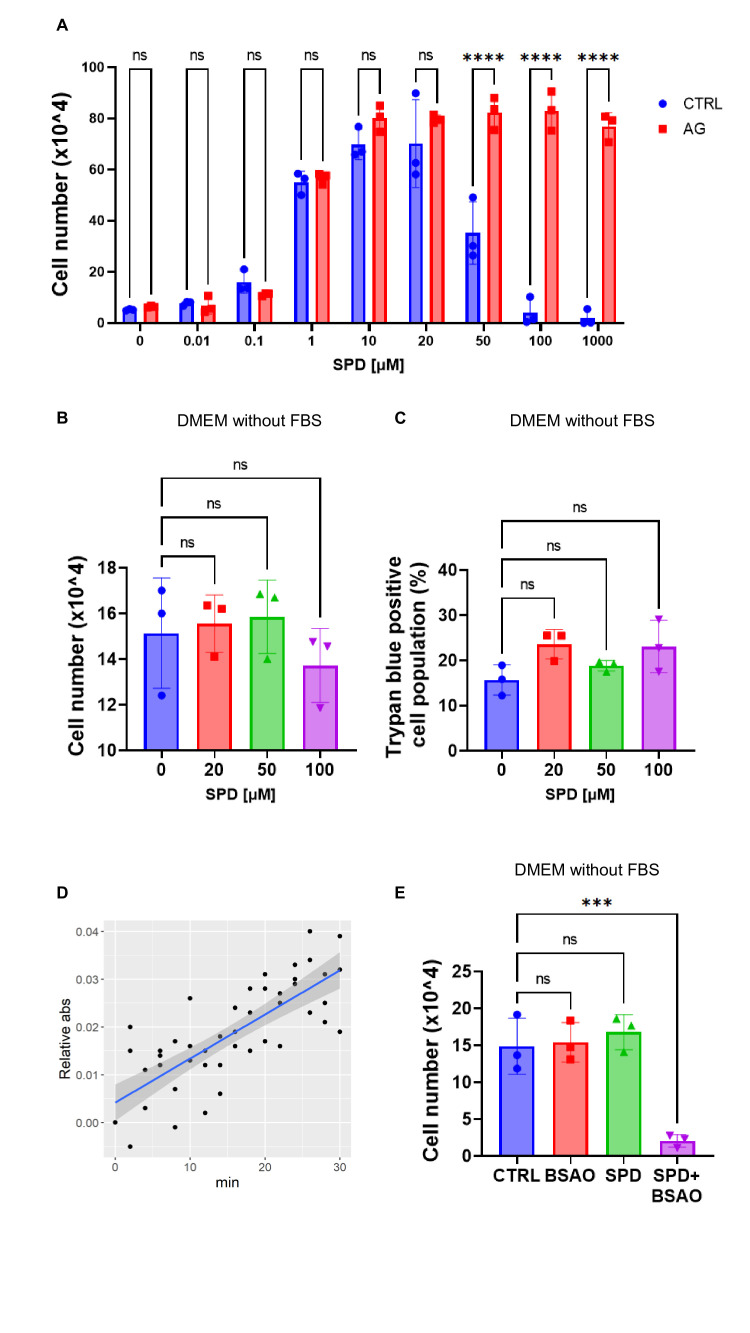
SPD-induced apoptosis is mediated by BSAO. (**A**) Cell proliferation assay of HCT116 cells (*n* = 3) treated with 500 μM aminoguanidine (AG) and with increasing concentrations of SPD (up to 1 mM) in DMEM containing FBS. Data represent the mean ± SD of experiments performed in triplicate. For statistical analysis, ****P < 0.0001 was determined by Šidák’s multiple comparisons test following two-way ANOVA. (**B**) Cell proliferation assay in HCT116 cells (*n* = 3) treated with different concentrations of SPD (0, 20, 50, and 100 µM) for 72 hours in DMEM without FBS. Data represent the mean ± SD of experiments performed in triplicate. For statistical analysis, ns = not significant (*P*>0.05) was determined using Dunnett’s multiple comparisons test following one-way ANOVA. (**C**) Percentage of HCT116 death cells (*n* = 3) assessed using the trypan blue exclusion assay after treatment with SPD as described in A. Data represent the mean ± SD of experiments performed in triplicate. For statistical analysis, ns = not significant (*P*>0.05) was determined using Dunnett’s multiple comparisons test following one-way ANOVA. (**D**) Enzyme kinetic assay to determine the amine oxidase activity of decomplemented FBS. Benzylamine was used as a substrate, and the amount of generated benzaldehyde (*ε* = 12, 500 M^−1^cm^−1^ at 250 nm, at 25°C) was quantified. The regression line was obtained from the data of three independent trials using a single regression model. The gray background shows the 95% confidence interval. (**E**) Cell proliferation assay in HCT116 cells (*n* = 3) treated with BSAO (1.5 × 10^−5^ U/ml) and 20 mM SPD for 72 hours in DMEM without FBS. Data represent the mean ± SD of experiments performed in triplicate. For statistical analysis, ns *P*>0.05, ***P<0.001 were determined using Dunnett’s multiple comparisons test following one-way ANOVA.

As documented in Figure 5A, inhibition of AO activity did not affect the proliferative effect of low doses of SPD, but it completely abrogated the cytotoxic effect observed with higher concentrations. To ascertain that the cytotoxic effect could be attributed to AO activity contained in the bovine serum, we tested the effect of SPD in medium lacking FBS. As shown in Figure 5B and C, SPD failed to inhibit cell proliferation and to induce cell death in the absence of FBS, as revealed by the unchanged number of trypan blue positive cells at all SPD concentrations tested.

To exclude the possibility that the toxicity could be related to components of FBS different from BSAO, we performed a kinetic assay of AO activity in FBS, measuring the rate of conversion of benzylamine to benzaldehyde. We observed that the AO activity was approximately ~1.5 ×10^−4^ U/ml (corresponding to 0.09 mmol/ml/hour), which is very close to 0.07 mmol/ml/hour reported in previous work using the same assay [22] (Figure 5D). Incubation of serum-deprived CRC cells with exogenous 1.5 × 10^−5^ U/ml BSAO and SPD caused a marked decrease in cell number compared with cells treated with individual agents or untreated (Figure 5E).

Together, these data demonstrate that AO activity of FBS is responsible for the SPD cytotoxicity.

### GC7 prevents both proliferative and cytotoxic effects of SPD by inhibiting DHPS and BSAO

Once established, the mechanism underlying the dual response to SPD, we tested the effect of GC7, a well-known inhibitor of DHPS [23], previously shown to possess significant anticancer properties in CRC cell culture, and in mouse models of intestinal tumorigenesis [7].

**Figure 6:**
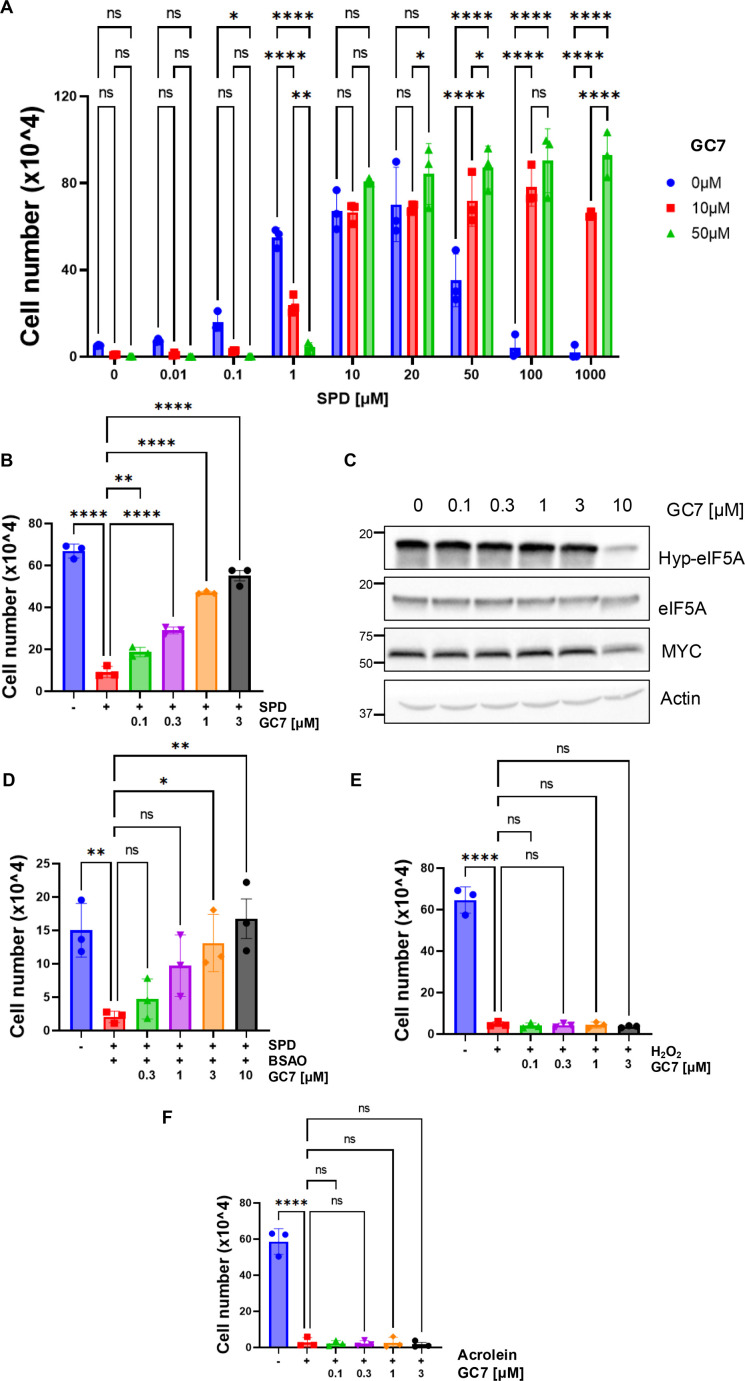
GC7 suppresses the cytotoxic activity of BSAO-metabolized SPD. (**A**) Cell proliferation assay in HCT116 cells (*n* = 3) treated with the indicated concentrations of SPD and GC7 for 72 hours in DMEM with FBS. Data represent the mean ± SD of experiments performed in triplicate. For statistical analysis, ns = not significant (*P*>0.05), *P<0.05, ****P<0.0001 were determined using Dunnett’s multiple comparisons test following two-way ANOVA. (**B**) Cell proliferation assay in HCT116 cells (*n* = 3) co-treated with 20 µM SPD and the indicated concentrations of GC7 for 72 hours in DMEM with FBS. Data represent the mean ± SD of experiments performed in triplicate. For statistical analysis, **P<0.01, ****P<0.0001 were determined using Dunnett’s multiple comparisons test following one-way ANOVA. (**C**) Western blot analysis showing hypusinated-eIF5A (Hyp-eIF5A), eIF5A, MYC, and Actin (loading control) in HCT116 cells treated with the indicated concentrations of GC7 for 48 hour. (**D**) Cell proliferation assay in HCT116 cells (*n* = 3) co-treated with 20 µM SPD, 1.5 × 10^−5^ U/ml BSAO and the indicated concentrations of GC7 for 72 hours in DMEM without FBS. Data represent the mean ± SD of experiments performed in triplicate. For statistical analysis, *P<0.05, **P<0.01 were determined using Dunnett’s multiple comparisons test following one-way ANOVA. (**E**) Cell proliferation assay in HCT116 cells (*n* = 3) co-treated with 80 µM hydrogen peroxide (H_2_O_2_) and with the indicated concentrations of GC7 for 72 hours in DMEM with FBS. Data represent the mean ± SD of experiments performed in triplicate. For statistical analysis, ns = not significant (*P*>0.05), ****P<0.0001 were determined using Dunnett’s multiple comparisons test following one-way ANOVA. (**F**) Cell proliferation assay in HCT116 cells (*n* = 3) co-treated with 30 µM acrolein and with the indicated concentrations of GC7 for 72 hours in DMEM with FBS. For statistical analysis, ns = not significant (*P*>0.05), ****P<0.0001 were determined using Dunnett’s multiple comparisons test following one-way ANOVA.

We treated the cells with 10 and 50 µM GC7, corresponding to doses commonly used to inhibit eIF5A hypusination, in polyamine-depleted cells treated with increasing amounts of SPD [8,18,24] (Figure 6A). As expected, GC7 prevented cell proliferation at low SPD doses in HCT116 and LoVo cells (Figure 6A and Supplementary Figure S3), reflecting the DHPS-mediated inhibition of eIF5A hypusination. Surprisingly, however, GC7 also reverted the cytotoxic effect of high doses of SPD in both cell lines tested, suggesting that it could also inhibit the enzymatic activity of BSAO. GC7 also abrogated the strong antiproliferative effect of SPD in cells grown without DFMO (Figure 6B). Indeed, by exposing HCT116 cells to 20 µM SPD and different concentrations of GC7, we observed a dose-dependent blockade of the antiproliferative effect that was fully abrogated with 3 µM GC7.

The effect could not be attributed to inhibition of DHPS because GC7 did not modify eIF5A hypusination at these dosages (Figure 6C). Likewise, GC7 prevented the antiproliferative effect of the combination 20 µM SPD+BSAO in a dose-dependent manner, and the rescue was complete at 3 µM (Figure 6D). By contrast, GC7 failed to revert the cytotoxic effect of H_2_O_2_ or acrolein (Figure 6E and F), demonstrating that its effect is exerted by counteracting BSAO-mediated oxidation of SPD but not by neutralizing its oxidation products.

### GC7 noncompetitively inhibits BSAO

Having established that GC7 alleviates the SPD cytotoxic effect by preventing its BSAO-mediated oxidation, we sought to understand the biochemical basis of this inhibition.

**Figure 7:**
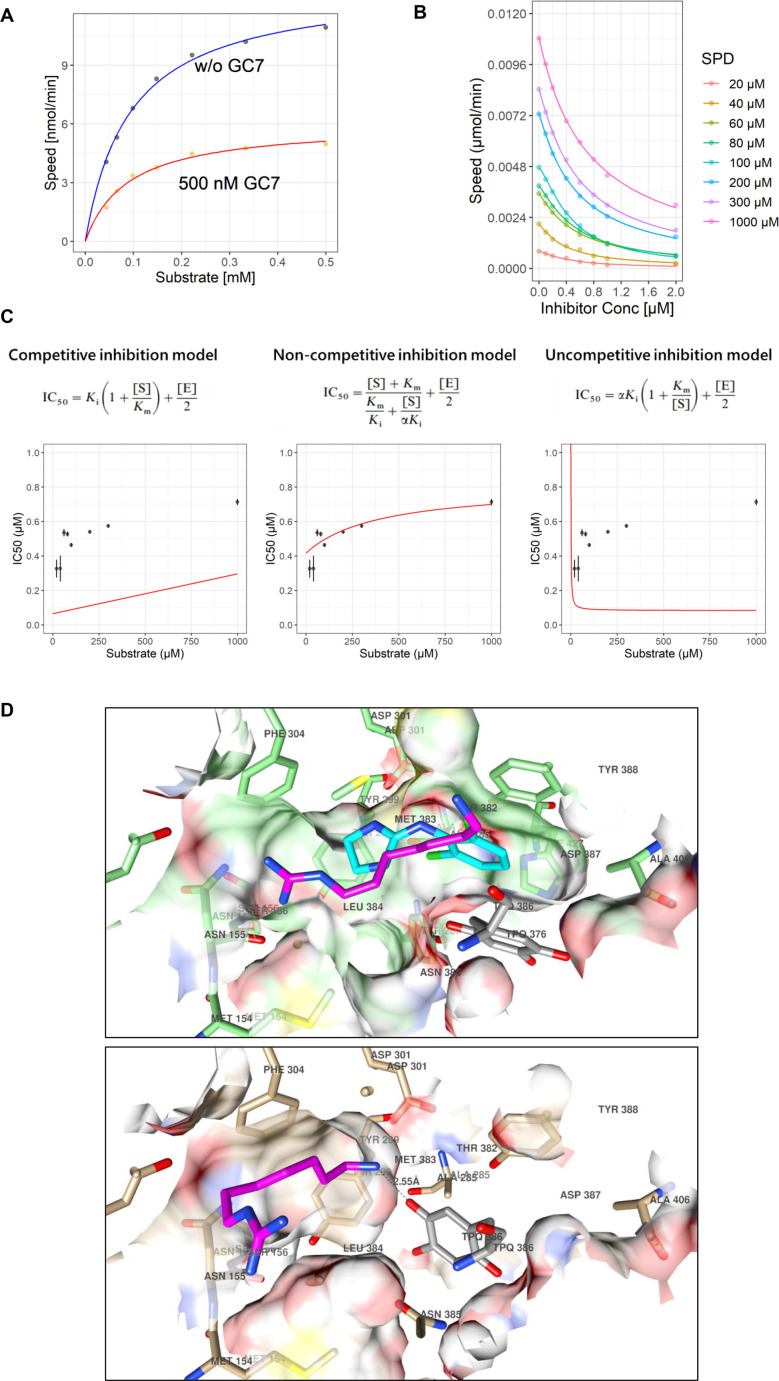
GC7 is a tight-binding inhibitor of BSAO. (**A**) Graph showing the relationship between BSAO enzymatic reaction speed (y-axis) and the concentration of substrate (x-axis) in the presence of 500 nM GC7 (red) or vehicle (blue). (**B**) Kinetic rates at increasing concentrations of inhibitor (GC7) for each substrate concentration (SPD) were measured using the DCHBS/AAP assay. Curve plots were constructed using a three-parameter log-logistic function. (**C**) IC50 and dissociation constants for the enzyme-inhibitor complex were calculated by fitting the dose-response data obtained in B using Morrison’s equation and formulae for calculating apparent Ki values depending on the mode of inhibition. Each red curve shows the fitting curve. AIC was used to compare each inhibition model. The red curves indicate the fittest curve under the conditions constrained by the apparent Ki equation and Morrison’s equation for each mode of inhibition. (**D**) Upper panel: GC7 (magenta) was docked into the inactive BSAO conformation. The conformation of the clonidine (cyan) complex is also depicted. The inactive conformation of TPQ is highlighted in gray. Bottom panel: GC7 (magenta) docked into the active BSAO conformation. The inactive conformation of TPQ is highlighted in gray.

We used the DCHBS/AAP (sodium 3,5-dichloro-2-hydroxybenzenesulfonate/4-aminoantipyrine) assay measuring the enzymatic reaction kinetics as an indicator of hydrogen peroxide production. First, as a pilot experiment, the reaction kinetics were measured using 100 nM (3.3 × 10^−4^ U/ml) BSAO and 500 nM GC7 with a maximum of 500 µM SPD as the substrate. As shown in Figures 7A and 500 nM concentration of inhibitor was sufficient to inhibit the activity of 100 nM enzyme. This result suggests that GC7 is a tight-binding inhibitor, because its inhibitory activity is observed even at concentrations that are not sufficiently high compared with the substrate concentration.

In the case of tight binding inhibitors, both competitive and non-competitive modes of inhibition showed similar patterns on the Lineweaver–Burk plot. Therefore, we calculated each kinetic parameter and selected the best-fitting model by nonlinear regression analysis using Morrison’s formula to analyze the inhibition data for tight-binding inhibitors and the formula for calculating each apparent inhibition constant (Ki). This method accounts for tight-binding conditions where inhibitor and enzyme concentrations are comparable, allowing robust discrimination of inhibition types and strengthening the interpretation of non-competitive inhibition. Data were obtained for the reaction rates at different GC7 combinations from 0.1 to 2 µM for substrate concentrations from 20 to 1000 µM (Figure 7B). With 20 μM SPD and 2 μM GC7, the reaction almost stopped, suggesting that GC7 can act as a complete inhibitor. The estimated parameters and IC50 values against the substrate concentrations are shown in Figure 7C. Of the three modes of inhibition, the non-competitive model best fits the estimates obtained from the measured values. In addition, Akaike’s information criterion (AIC) values, which estimate the goodness of fit of a model, were 546.1792, −891.2711, and −517.7613 in the competitive, non-competitive, and uncompetitive models, respectively. These results indicate that GC7 is a non-competitive inhibitor. Considering Km, Vmax, alpha, Ki, [E] as parameter kinetics, the best-fit values (±S.E.) resulted to be Km = 173.8 (±16.35) μM; Vmax = 12.98 (±0.505) nmol/minute; alpha = 2.28419 (±0.30153); Ki = 0.30775 (±0.03711) μM; [E] = 0.218 (±0.09025) μM, respectively.

### *In silico* docking analysis revealed that GC7 binds to BSAO independently of TPQ conformation

Molecular docking (MDock) [25] to free BSAO (1TU5) was performed to confirm the ability of the software to find low-energy poses close to those observed in the inhibited protein (2PNC). This reaction mechanism likely depends on the conformational state of trihydroxyphenylalanine quinone (TPQ). Indeed, the two different experimental BSAO side chains in the TPQ orientations indicate the *off-copper* and on-*copper* states [26] as found in 1TU5 and 2PNC, respectively. Regardless of the TPQ orientation, MDock simulations were performed on polyamine analogs using classical reversible molecular docking (RMDock) procedures.

As available within the 3d-qsar.com portal, the programs Smina [27] and Plants [28] were used to assess which software/scoring function combination would result in a lower average root mean square deviation (RMSD) value. In fact, the cross-docking of clonidine (CLO) into free BSAO showed a high RMSD value, indicating that the drug was not able to correctly bind to the off-copper BSAO conformation (Supplementary Table S1). In contrast, the RMSD values of redocked CLO in the copper BSAO conformation confirmed the ability of the programs to reproduce, to some extent, the binding conformation of CLO. In particular, the Plants/PLP program/scoring function combination was the most effective, with average RMSD values of 1.56 and 0.62. From the re-docking experiments, it was also evident that CLO was much better repositioned in chain A of 2PNC than in chain B.

As the Plants/PLP program/scoring function combination was the most effective, it was thus applied as a tool to investigate the unbound conformation of the titled polyamines into the four BSAO protein chains extracted from 1TU5 and 2PNC.

GC7 was docked into either BSAO TPQ *off-copper* or *on-copper* state conformations, as experimentally reported in the Protein Data Bank (PDB) with the codes 1TU5 and 2PNC, respectively.

Docking of the GC7 conformation into inhibited BSAO (2PNC) revealed that the compound fully overlapped with the complexed CLO inhibitor (Figure 7D). The docked conformation of GC7 was found to be buried in a pocket formed by the main chains and side chains of Met154, Asn155, Ser156, Tyr299, Asp301, Phe304, Ser371, Thr372, Met373, Leu374, Asp377, and Tyr378, while forming van der Waals and electrostatic interactions. In particular, the GC7 guanidine moiety appeared to establish a hydrogen bond with the carbonyl oxygen of Asn155 (distance 2.66 Å). The docking of GC7 into the free BSAO conformation revealed a slightly different binding mode, likely due to the different TPQ conformations that pushed the compound into a pocket formed by Tyr119, Asn155, Tyr182, Tyr299, Asp301, Phe304, Phe309, Thr382, Met383, Leu384, and Tyr388 (Figure 7D). The compound mainly established van der Waals interactions with no particular hydrogen bonding, but interestingly, the terminal amino group was placed at 2.55 Å from the TPQ oxygen at position 5 (the reactive one for the Schiff base formation).

Inspecting the docked energies (Supplementary Table S2) revealed that GC7 binds with slightly lower energy [29] to the inhibited BSAO (2PNC) than to the free one (1TU5), suggesting that the preferred BSAO conformation would be that one. Alternatively, as the score energy difference is not very different, it could be speculated that GC7 is able to bind to both BSAO conformations, which is supported by the observation that the orientation of the docked GC7 is maintained in either BSAO conformation.

## Discussion

SPD has recently garnered considerable attention as a natural compound with diverse health-promoting effects, including lifespan extension, cardioprotection, neuroprotection, anti-inflammatory properties, and tumor suppression [30–33]. Mechanistically, these effects have been attributed to SPD’s ability to induce autophagy, either via P300-mediated histone acetylation or through eIF5A hypusination, which promotes the translation of autophagy-related targets such as TFEB and ATG3 [18,34]. These insights have fueled interest in using SPD-rich diets or supplements to prevent or mitigate age-related diseases. Supporting this idea, SPD supplementation has shown promising safety profiles and reduced cognitive decline in elderly populations during clinical trials [35]. In 2019, the European Union approved the commercialization of SPD-rich wheat germ extract from Longevity Labs, underscoring its potential as a dietary supplement.

Recently, SPD supplementation has also been reported to mitigate intestinal tumorigenesis in APC-mutated mice [13], suggesting its possible use in patients with FAP or in individuals at risk of CRC onset or recurrence.

However, the well-established role of elevated intra-tumoral polyamine levels in driving cancer growth complicates the narrative. Indeed, while SPD may combat aging and prevent certain diseases, concerns remain regarding its potential to promote tumorigenesis.

Many studies have investigated the mechanism of action of SPD using cultured cells exposed to various concentrations of this polyamine. While some reports have documented that exogenous SPD induces cell proliferation [11,32,36–38], others have documented its effects on autophagy [17,39,40], while others have shown cytotoxicity [20,41,42].

**Figure 8:**
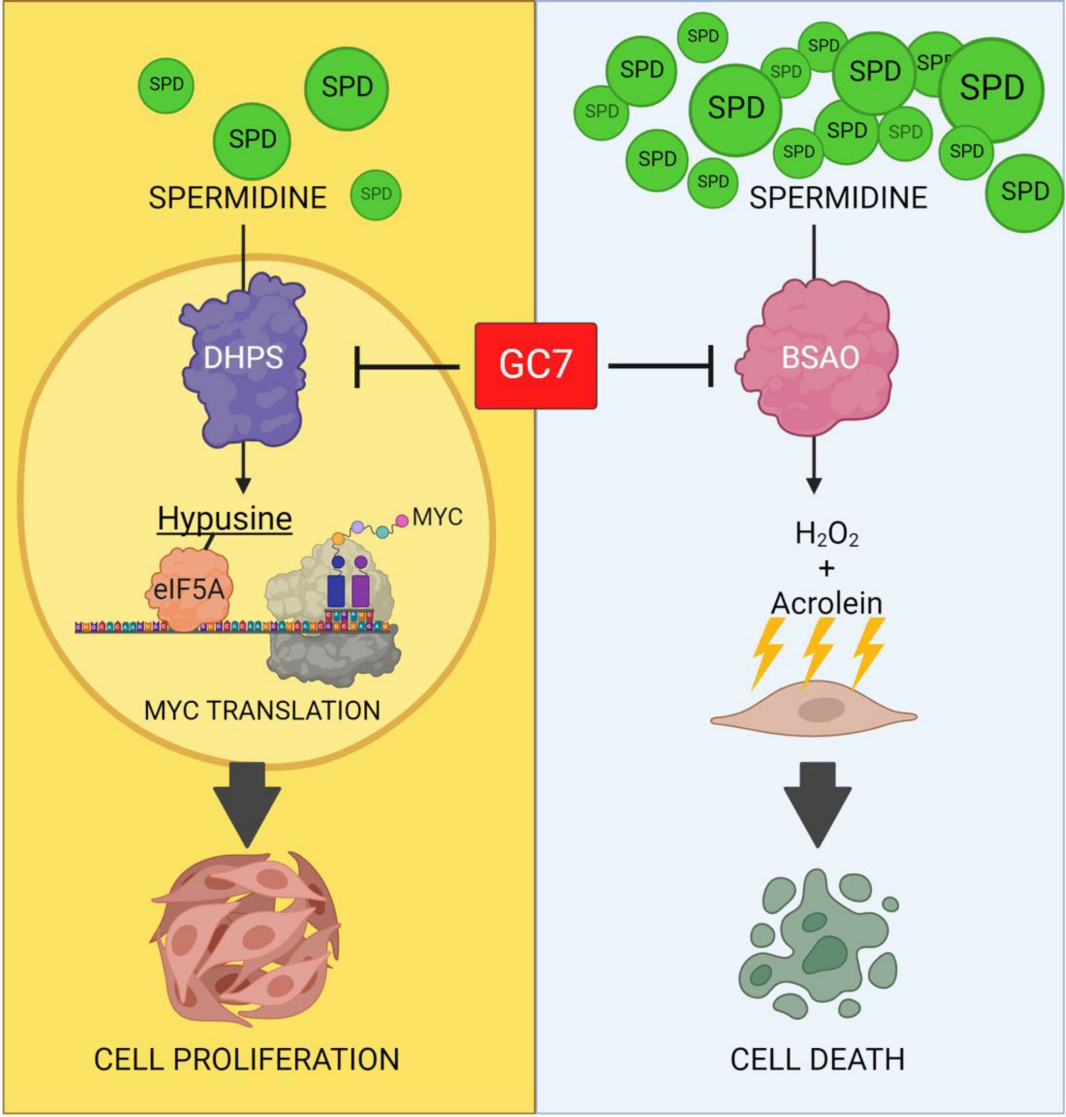
Working model of SPD low and high doses in CRC cells. In CRC cells, low doses of SPD (1–10 µM) trigger the DHPS-mediated hypusination of eIF5A, promoting MYC translation and cellular proliferation. At high SPD doses (>50–100 µM), the BSAO activity is predominant and promotes the release of H_2_O_2_ and acrolein from SPD oxidation, causing oxidative stress and DNA damage and leading to cell death. GC7 acts as a dual inhibitor of eIF5A hypusination, preventing cell proliferation, and of BSAO preventing cell death.

To reconcile all these observations into a unifying model, here we have examined the effects of a range of SPD concentrations in cell culture models deprived of exogenous polyamines. Our findings revealed a dose-dependent, dual effect of SPD on cancer cell proliferation and viability. At low concentrations (1–10 µM), SPD promoted DHPS-mediated eIF5A hypusination, MYC translation, and tumor cell proliferation. Conversely, at higher concentrations (>50–100 µM), SPD was oxidized by BSAO present in the FBS, and these events led to the production of cytotoxic byproducts that negated the eIF5A-mediated proliferative response and induced cell death (Figure 8).

It would be important to determine whether the cytotoxic effects of SPD mediated by BSAO *in vitro* also occur *in vivo* and to clarify the potential translational relevance of these data. It is conceivable that SPD-induced cytotoxicity may occur *in vivo* through the activity of endogenous AOs. Humans express semicarbazide-sensitive AO vascular adhesion protein-1 (VAP-1), which shares substrate specificity with BSAO and could, in principle, mediate SPD oxidation within specific tissues or tumor microenvironments [–45]. This suggests that the cytotoxic and growth-suppressive effects observed in our study may have physiological relevance, particularly in contexts where AO expression is elevated. Future work should investigate AO expression across human tumor types and assess whether targeted delivery of high-dose SPD, such as via nanoparticle formulations, could exploit localized AO activity to induce selective cancer cell death. The above-described report showing reduced tumor burden in APC^min^ mice following oral SPD administration [13] is consistent with the idea that *in vivo* SPD oxidation may contribute to its antitumor activity. These observations point to potential translational applications of high-dose SPD, either as a single agent or in combination with therapies that augment oxidative stress or impair redox detoxification pathways. Nevertheless, further mechanistic studies are needed to delineate the contribution of endogenous AOs to SPD-mediated cytotoxicity and to determine whether this pathway can be harnessed therapeutically without inducing off-target toxicity.

A particularly contentious topic is whether SPD induces autophagy. Previous studies by the R. Casero group suggested that SPD-induced autophagy is caused by cytotoxic byproducts of BSAO-mediated SPD oxidation [46]. Their experiments demonstrated that the BSAO inhibitor AG abolished autophagic responses, arguing against the direct roles of SPD in autophagy through inhibition of intracellular targets such as eIF5A or P300. These conclusions, based on experiments using 25 µM SPD, align with our findings, as we also observed cytotoxicity at similar SPD concentrations in cells not pretreated with DFMO. However, those studies evaluated autophagy only under higher SPD concentrations and at a single 6 hour time point, leaving open the possibility of autophagy induction under alternative conditions, such as prolonged exposure to lower SPD doses that primarily activate the DHPS-eIF5A axis. To explore this, we assessed autophagic responses in polyamine-depleted cells treated with both low and high SPD doses over short and long durations. Our results indicate that lower SPD concentrations induce eIF5A hypusination but do not modify autophagy at any time points, suggesting that the pro-tumorigenic activity observed under those conditions cannot be attributed to a modulation of the autophagic flux. Conversely, higher SPD doses inhibit the autophagic flux at the autophagosome stage after short-time incubation (6 hours), but not at longer time points (24 hours), indicating that the inhibition of autophagy is a transient event that likely reflects the cellular response to the oxidative stress induced by BSAO-mediated SPD oxidation.

A significant finding of this study is the identification of GC7 as a potent inhibitor of BSAO (Figure 8). GC7, a well-established competitive inhibitor of DHPS, has been widely used to block eIF5A hypusination and inhibit tumor growth *in vitro* and *in vivo*, including in CRC models [7]. However, questions about GC7’s exclusive specificity for DHPS have persisted [47], raising the possibility of additional targets. Our kinetics and *in silico* analyses revealed that GC7 acts as a tight-binding, non-competitive inhibitor of BSAO. Docking studies indicated that GC7 interacts with BSAO independently of the structural conformation of its TPQ organic cofactor, and kinetic studies confirmed that GC7’s inhibition of BSAO is reversible. Supporting this, BSAO activity was restored following dialysis of samples containing GC7, SPD, and BSAO (not shown). Therefore, our findings reinforce that phenotypes ascribed to DHPS inhibition on the basis of GC7 treatment alone should be interpreted with caution, owing to the compound’s BSAO-mediated off-target effects. To strengthen causal links to DHPS/eIF5A, complementary strategies such as genetic DHPS depletion or the use of non-hypusinatable eIF5A mutants can provide more specific mechanistic insight. Moreover, suppressing BSAO activity, for instance, using AG or serum-free/defined media, helps disentangle DHPS-dependent effects from additional GC7 effects. Together, these approaches can better discriminate *bona fide* DHPS/eIF5A-driven phenotypes from GC7-related artifacts.

In conclusion, these findings highlight the dual roles of SPD and GC7 in targeting distinct enzymes with opposing effects. SPD promotes tumor cell proliferation at low doses via DHPS and eIF5A hypusination, while high doses are cytotoxic due to BSAO-mediated oxidation. GC7, on the other hand, inhibits DHPS to suppress proliferation and blocks BSAO to mitigate SPD-induced cytotoxicity.

This study underscores the need for careful evaluation of SPD and GC7 dosages and conditions before their use in *in vitro* settings. Future research should focus on defining the dose-response relationships and mechanism of action in pathophysiologically relevant study models.

## Materials and methods

### Cell culture and treatments

HCT116 (ATCC; CCL-247) and LoVo (ATCC; CCL-229) cells were grown in Dulbecco's Modified Eagle's Medium (DMEM) supplemented with 10% FBS, penicillin (100 IU/ml), streptomycin (100 μg/ml), and 2 mM L-glutamine purchased from SIGMA Aldrich. For each passage, which was performed every 2 or 3 days, confluent cells were harvested with 10 mM EDTA in PBS, followed by the further addition of 0.1% trypsin solution dissolved in PBS to detach cells. Proliferation assays were performed by seeding 10^4^ or 2 × 10^4^ cells/cm^2^ and counting their number with a Burker chamber at the indicated time points, after staining with Trypan Blue (Sigma Aldrich #T6146). SPD and DFMO treatments were performed using SPD purchased from SIGMA Aldrich (#S2626) and DFMO purchased from SIGMA Aldrich (#D193). AMXT-1510 was purchased by Targetmol (#T10313).

### Flow cytometry

SPD-induced apoptosis in polyamine-depleted HCT-116 cells was evaluated by flow cytometry following the manufacturer’s instructions using APC Annexin V (#550475) and 7-AAD (#559925), purchased from BD Pharmingen. After pre-incubation with 1 mM DFMO to induce polyamine depletion, cells were seeded back in a 60 mm dish plate at a density of 5 × 10^4^ cells/cm^2^ in DMEM supplemented with FBS and treated with SPD (20 μM or 1 mM) and DFMO for 48 hours at 37°C. After treatment, cells were harvested and stained. The intensity of fluorescence emitted from the stained cells was analyzed on the FL-1 channel (533/30 nm) and the FL-3 channel (647/nm), respectively, with an excitation at 488 nm. At least 10,000 events/samples were acquired, using a FACSCalibur (BD).

### Western blotting analysis

Western blot analysis was performed as previously described [48]. Cells were washed with PBS, collected, and lysed in RIPA buffer (50 mM Tris-HCl pH 7.6; 0.5% deoxycholate; 150 mM NaCl; 1% Triton-100; 0.1% SDS; 2 mM EDTA pH 8.0) containing SIGMAFAST™ Protease Inhibitor Tablets (AEBSF 20 μM, EDTA 10 μM, bestatin 1.3 μM, E-64 140 nM, leupeptin 10 nM, and aprotinin 3 nM) (Sigma-Aldrich #S8820). Cell lysates were centrifuged at 13,000 rpm at 4°C for 30 minutes, and the supernatants were collected. Protein concentration was determined using the Bradford assay reagent (Bio-Rad #5000006). Cell lysates were mixed with 4× Laemmli sample buffer (Bio-Rad #1610747) containing 10% β-mercaptoethanol (Thermo Fisher #M3148) and boiled for 5 minutes. Protein samples were separated by SDS-PAGE and then transferred onto a nitrocellulose membrane using a Turboblot transfer kit (Bio-Rad). Membranes were blocked in 5% skim milk in TBS-T (Tris-Base 20 mM, NaCl 137 mM, pH 7.5, 0.08% Tween-20) for 30 minutes and incubated with primary antibodies overnight at 4°C with gentle agitation. Membranes were then incubated with horseradish peroxidase (HRP)-conjugated secondary antibodies (Bio-Rad #1706515) at room temperature for 1 hour. Besides the indirect detection method, HRP-conjugated anti-β-actin antibody (Thermo Fisher #MA5-15739-HRP) and HRP-conjugated anti-vinculin antibody (SantaCruz #sc-73614HRP) were used at a 1:10,000 dilution to detect the housekeeping protein. After incubation, the filters were washed with TBS-T and exposed to a Western ECL solution (Bio-Rad #1705061) for 5 minutes. Chemiluminescence was determined using an iBright™ Imaging System (Thermofisher).

Total eIF5A antibody (#ab32443) and DHPS antibody (#ab202133) were purchased from Abcam. MYC antibody (9402S) was purchased from Cell Signaling Technology. Hyp-eIF5A antibody was kindly provided by Professor Raghu Mirmira, Indianapolis, U.S.A. [49].

Primary antibodies were diluted in 1:1000 with 5% skim milk in TBS-T, except for MYC (1:2000), eIF5A (1:5000), and hyp-eIF5A (1:5000) antibodies.

### RNA knockdown

For viral production, HEK-293T cells were transfected with PMD2.G (Addgene #12259), PCMVR8.74 (Addgene #22036), and pLKO.1 vectors using calcium phosphate precipitation as previously described [50]. The sequences of the inserted oligonucleotides are:

shDHPS FW 5´-CCGGAGTGCACTGGGATGATCATTCTCTAGAGAATGATC-3´

shDHPS RW 5´-AATTCAAAAAAGTGCACTGGGATGATCATTCTCTAGAGA-3´

sheIF5A FW  5´-CCGGGCATTACGTAAGAATGGCTTTTCTAGAAAAGCCAT-3´

sheIF5A RW 5´-AATTCAAAAAGCATTACGTAAGAATGGCTTTTCTAGAAA-3´

Mission plko.1-puro scramble shRNA (Sigma-Aldrich, #SHC002) was used to produce the control virus expressing non-targeting shRNA, and we named it shSCR within the text. After 24 hours, DMEM was replaced. On the following day, the supernatant was harvested, centrifuged at 1,000 rpm for 5 minutes at 25°C, and filtered through a 0.45 mm pore. HCT116 cells dispersed in DMEM were transduced with lentiviruses, and the culture media was changed after 24 hours. After 72 hours, cells with puromycin (5 μg/ml) were added for an additional 72 hours to select transduced cells.

### LC3B-GFP-mCHERRY puncta formation assay

The plasmid pCDH-CMV-mC-G-LC3B-P (Addgene, #124974) was used for lentiviral production. This plasmid, along with the packaging plasmids PMD2.G (Addgene, #12259) and PCMVR8.74 (Addgene, #22036), was transfected into HEK-293T cells. HCT116 and LoVo were transduced with the generated lentivirus for 72 hours and selected by supplementing the culture medium with 5 μg/ml puromycin for an additional 72 hours. Selected cells were seeded in u-SLIDE 8 WELL ibiTreat chambers (IBIDI, #80826). Cells were pre-treated with 1 mM DFMO for 72 hours and subsequently treated with SPD at the indicated concentrations for either 6 or 24 hours. Live-cell imaging was performed using a ZEISS LSM 980 with Airyscan 2 laser-scanning confocal microscope, employing a 63 × oil immersion objective. Scale bars were applied in Fiji 2.14.0 using the metadata collected during image acquisition.

### Lysotracker Red staining

Lysotracker Red was purchased by Medchem Express (#HY-D1300) and used according to the manufacturer’s specifications. Briefly, cells were seeded in u-SLIDE 8 WELL ibiTreat chambers (IBIDI, #80826), pre-treated with 1 mM DFMO for 72 hours, and subsequently treated with SPD at the indicated concentrations for either 6 or 24 hours. Before imaging, cells were treated with 50 nM Lysotracker Red for 30 minutes and 1X Hoechst 33,342 for 10 minutes. Living cells were imaged using a ZEISS LSM 980 with Airyscan 2 laser-scanning confocal microscope, employing a 63× oil immersion objective. Scale bars were applied in Fiji 2.14.0 using the metadata collected during image acquisition.

### EdU incorporation assay and immunofluorescence

The Click-iT EdU Alexa Fluor 488 HCS Assay (Invitrogen, #C10350) was performed following the manufacturer’s protocol to assess cell proliferation. Specifically, cells were supplemented with EdU for 6 hours prior to fixation with 3.7% formaldehyde in PBS. Permeabilization was achieved using 0.5% Triton X-100 in PBS. The Click-iT reaction cocktail was prepared as specified by the manufacturer and incubated for 30 minutes.

For immunofluorescence staining, non-specific binding was blocked with 3% bovine serum albumin in PBS for 30 minutes. Cells were then incubated overnight at +4°C with the primary antibody against cleaved caspase-3 (Cell Signaling, #9661) diluted 1:250. The following day, cells were incubated for 30 minutes with the secondary antibody, Goat anti-Rabbit Alexa Fluor™ 594 (#A-11012), diluted 1:800. Finally, nuclei were counterstained by adding 1X Hoechst 33,342 solution for 10 minutes.

Confocal images were acquired using a ZEISS LSM 980 with Airyscan 2 laser-scanning confocal microscope, employing a 63 × oil immersion objective. Scale bars were applied in Fiji 2.14.0 using the metadata collected during image acquisition.

### Purification of BSAO

Bovine blood was withdrawn at a processing facility and mixed with 3.8% sodium citrate solution (an anticoagulant), and then the copper-enzyme BSAO was isolated by performing a combination of ionic exchange and affinity chromatographic techniques, as previously reported [51,52]. All purification steps were performed in a cold room, at 4°C. The enzyme purification factor was approximately 1600-fold, and a single band was observed on 6% SDS-PAGE gel.

The protein concentration for the enzyme was assayed by the absorbance at 280 nm, using an absorption extinction coefficient of 1.74 L g^−1^ cm^−1^. BSAO and decomplemented FBS activities were determined spectrophotometrically as the amount of benzaldehyde formed per min (μmol/min) from benzylamine at 250 nm (*ε* = 12,500 M^−1^ cm^−1^) in 0.1 M sodium phosphate buffer (pH 7.2) at 25°C.

### Enzyme kinetic assay

The inhibitory effect of GC7 was examined by DCHBS/AAP assay. The BSAO catalytic properties were spectrophotometrically determined by detecting the amount of hydrogen peroxide formed during the oxidative deamination of the polyamines spermine or SPD. In the presence of H_2_O_2_, DCHBS is oxidized by HRP to a semiquinone radical form, which generates a pink quinonimine derivative reacting with AAP. When a solution of 1 mM AAP and 2 mM DCHBS was present, the determinations were performed in 0.1 M PBS buffer (pH 7.4) at 37°C, using SPD as a substrate to determine BSAO activity [53].

The pink adduct (ε_515_ = 26,000 M^−1^ cm^−1^), generated by the oxidation of the polyamine, was detected spectrophotometrically using NP-40. The kinetic constants, both affinity constant ( $K_{m}$ ) and maximum reaction speed ( $V_{max}$ ), values, were estimated using a non-linear regression model of the Michaelis–Menten equation.

The following Morrison’s equation was applied to examine the mode of tight-binding inhibition [54].


$$\frac{v_{i}}{v_{0}}=1-\frac{([E]+[I]+K_{i}^{app})-\sqrt{{\left([E]+[I]+K_{i}^{app}\right)}^{2}-4[E][I]}}{2[E]}$$

Where $v_{0}$ is the maximum reaction velocity and $v_{i}$ is the reaction velocity in each condition. $\left[E\right]$ and $\left[I\right]$ means the concentration of isolated enzyme and inhibitor, respectively. The value $K_{i}^{app}$ is related to the true $K_{i}$ (the inhibitor constant given by $K_{i}=\frac{\left[E\right]\left[I\right]}{\left[EI\right]}$ ) by factors involving the substrate concentration and $K_{m}$ , depending on the mode of interaction between the inhibitor and the enzyme.

The explicit forms of $K_{i}^{app}$ for the different inhibitor types are as follows:

For competitive inhibitors: $K_{i}^{app}=K_{i}\left(1+\frac{\left[S\right]}{K_{m}}\right)$

For non-competitive inhibitors: $K_{i}^{app}=\frac{\frac{[S]+K_{m}}{K_{m}}}{\frac{K_{m}}{K_{i}}+\frac{\left[S\right]}{\alpha K_{i}}}$

For uncompetitive inhibitors: $K_{i}^{app}=K_{i}\left(1+\frac{K_{m}}{\left[S\right]}\right)$

$V_{max}$ and $K_{m}$ obtained from the Michaelis–Menten equation were assigned to estimate the inhibition type and parameters by nonlinear regression analysis. AIC was calculated to compare each inhibition model [55].

#### *In silico* docking

The molecular modeling procedure has been worked out as recently reported [55]. GC7 was modeled through the free for academic MolEdit molecular editor (https://www.molsoft.com/moledit.html) embedded in the www.3d-qsar.com Py-MolEdit web app [56]. Canonical SMILES structures were obtained with a simple Python script using OpenBabel APIs [57].

The BSAO structures available from PDB, 1TU5, and 2PNC were processed through the www.3d-qsar.com Py-PDB web app. The structures were retrieved and cleaned up of all ions and molecules that were not involved in the catalytic process. Together with the standard amino acid residues, the TPQ and the catalytic copper ion were retained. The cleaned structures were then added to the hydrogens considering a pH of 7.6 and subjected to a short energy minimization (100 iterations of steepest descent and 50 iterations of conjugated gradient) with the universal force field (UFF) [58], as implemented in the OpenBabel suites. The BSAO apoproteins and the CLO ligands were saved separately in a dataset container.

Through the Py-Docking web app available at www.3d-qsar.com, two docking programs, Smina [27] and Plants [28], were applied to the 1TU5 and 2PNC BSAO complexes, considering all the implemented scoring functions (vina, vinardo, and ad4 for Smina, and PLP, PLP95, and ChemPLP for Plants). Re-docking and cross-docking assessment experiments were run directly through the www.3d-qsar.com server. GC7 was then docked using the most appropriate program/scoring function combination (see results section in the main text) saving in a separate dataset container the lowest docked conformations for each derivative.

The GC7 starting conformation was generated from the SMILES structure. The SMILES was converted to a 3D conformation and refined with a local minimization using the UFF force field available in the OpenBabel program.

The default settings were used for the dockings, except for setting a cubic box extending 30 Å centered at the CLO center of mass. For Smina, an exhaustiveness of 32 was set, and the search_speed key was set to 1 for Plants.

All computations for the 3-D QSAR and COMBINE model generation were run on the 3d-qsar.com portal (https://www.3d-qsar.com/) freely available to anyone for non-profit usage, designed and maintained by the authors. All other used stand-alone or command-line software was free and publicly available: UCSF Chimera (https://www.cgl.ucsf.edu/chimera/download.html); Anaconda was used as Python environment (https://www.anaconda.com/products/distribution) with the free and open-source available libraries (RDKit - https://www.rdkit.org/; OpenMM - https://openmm.org/; scikit-learn - https://scikit-learn.org/stable/ and OpenBabel - http://openbabel.org/). The co-crystallized structure of BSAO, one co-crystallized with the inhibitor CLO [59].

(CLO, PDB entry code 2PNC) and the free unbound BSAO protein [60] (PDB entry code 1TU5), was employed to select the docking software through a reversible docking assessment procedure. Furthermore, cross-docking experiments [61,62] were also performed as the BSAO-bound TPQ cofactor [52,63] was observed to have two distinct side chain conformations in either the CLO-bound (2PNC) or the free structure (1TU5).

The MDock [25] into free BSAO (1TU5) was performed to confirm the software’s ability to find low-energy poses close to those observed in the inhibited protein (2PNC). Regardless of the TPQ orientation, MDock simulations were carried out on polyamine analogs by means of classical RMDock procedures.

As available within the 3d-qsar.com portal, the programs Smina [27] and Plants [28] were used to assess which software/scoring function combination would result in a lower average RMSD value. The MDock results files are available at the Figshare link: 10.6084 /m9.figshare.29382365.

#### Statistical analysis

Statistical analyses were conducted using GraphPad Prism, version 9.2 (GraphPad, San Diego, CA, U.S.A.) or with the R package ggplot2. *P*-values were obtained from one-way or two-way ANOVA followed by post hoc analysis as specified in the figure legends for each experiment. Data are presented meeting the assumptions of independence of observations, normality, and homogeneity of variance for each experimental group tested. Statistical significance was determined as *P*<0.05.

## Supplementary material

online supplementary figure 1.

## Data Availability

Data presented in this paper will be made available upon request to the corresponding authors. Uncropped Western blots are included as Supplementary Data. The molecular docking results files are available at the Figshare link: 10.6084/m9.figshare.29382365 [64].

## Open Access

This article has been published open access under our Subscribe to Open programme, made possible through the support of our subscribing institutions, learn more here: https://portlandpress.com/pages/open_access_options_and_prices#conditional

## Abbreviations

AG: aminoguanidine
AIC: Akaike’s information criterion
AO: amine oxidase
BSAO: bovine serum amine oxidase
CLO: clonidine
CRC: colorectal cancer
DCHBS/AAP: sodium 3,5-dichloro-2-hydroxybenzenesulfonate/4-aminoantipyrine
DFMO: difluoromethylornithine
DHPS: deoxyhypusine synthase
DMEM: Dulbecco's Modified Eagle's Medium
EdU: 5-ethynyl-2′-deoxyuridine
FAP: familial adenomatous polyposis
FBS: fetal bovine serum
HRP: horseradish peroxidase
MDock: molecular docking
ODC: ornithine decarboxylase
RMDock: reversible molecular docking
RMSD: root mean square deviation
SPD: spermidine
TPQ: trihydroxyphenylalanine quinone
UFF: universal force field
eIF5A: eukaryotic initiation factor 5A
