# Supplementary material for: Opposite effects of spermidine and GC7 in cell culture are dictated by distinct molecular targets
Source: Biochem J. 2025 Dec 17;482(24):1973–92. doi: 10.1042/BCJ20253298 (PMC12802350; doi:10.1042/BCJ20253298)
Supplement: online supplementary figure 1. [file bcj-482-24-BCJ20253298-s001.pdf]

Supplemental Fig. 1. SPD induces cell death at lower concentrations in cells without polyamine-depletion

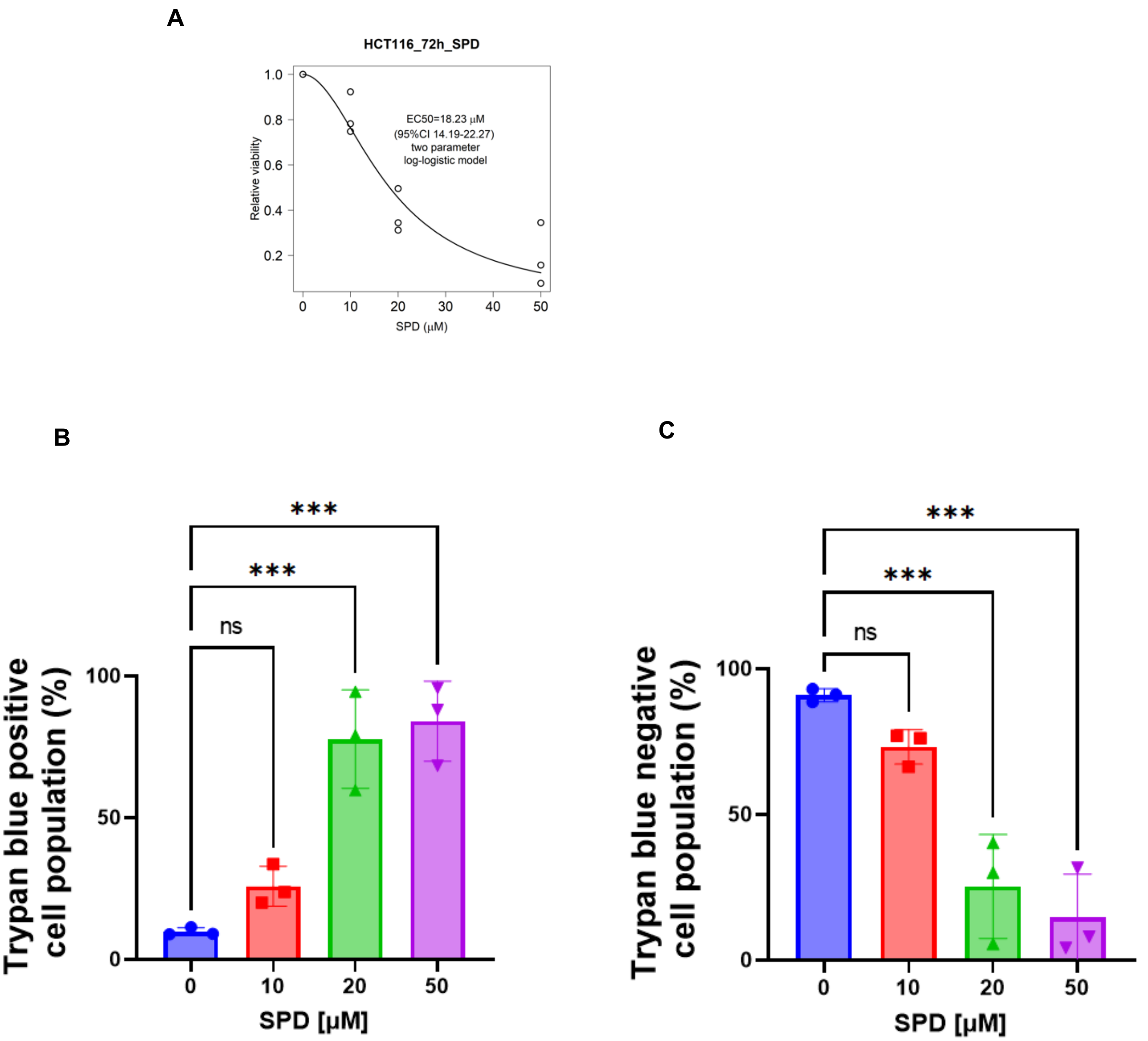

Supplemental Fig. 1:

(A) Effect of increasing concentrations of SPD in HCT116 cells without pre-treatment with DFMO.

(B) Percentage of Trypan blue positive (death) HCT116 cells (n=3) assessed using the trypan blue exclusion assay after treatment with SPD, as described in A. Data represent the mean ± SD of experiments performed in triplicate. For statistical analysis, ns=not significant (p>0.05), *p < 0.05 were determined using Dunnett's multiple comparisons test following One-way ANOVA.

(C) Percentage of Trypan blue negative (viable) HCT116 cells (n=3) assessed using the trypan blue exclusion assay after treatment with SPD as described in A. Data represent the mean ± SD of experiments performed in triplicate. For statistical analysis, ns=not significant (p>0.05), *p < 0.05 were determined using Dunnett's multiple comparisons test following One-way ANOVA.

Supplemental Fig. 2. SPD does not induce autophagy in cells without polyamine-depletion.

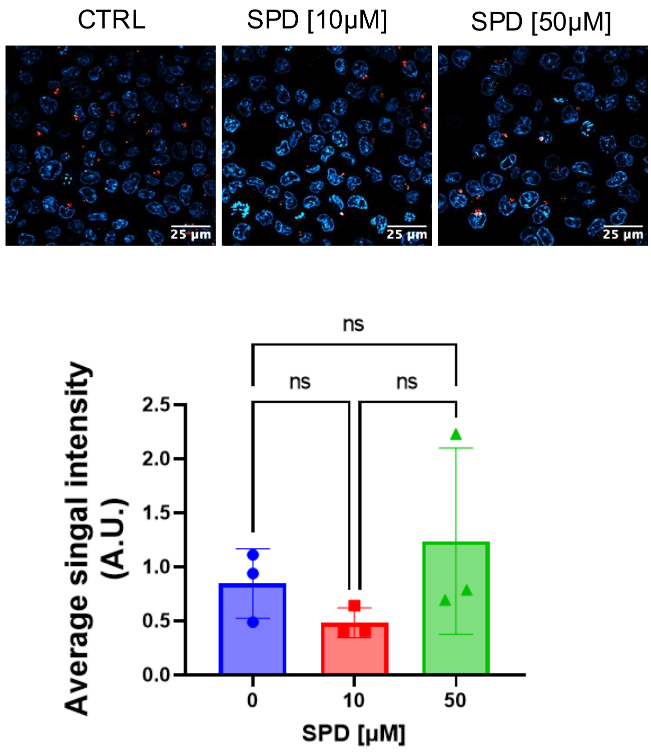

Supplemental Fig. 2:
Upper panel: Representative confocal images of HCT116 cells treated for 24 hours with 0, 10 or 50 µM SPD. Before imaging, live cells were stained with Lysotracker Red (red puncta) and DAPI (blue). Scale bar 40µm. Bottom panel: Quantification of Lysotracker Red signal using ImageJ from 3 independent images normalized to the respective cell number. For statistical analysis, ns=not significant ($p>0.05$) was determined by Dunnett's multiple comparisons test following One-way ANOVA.

Supplemental Fig. 3. GC7 suppresses the cytotoxic effect of high doses spermidine in LoVo cells

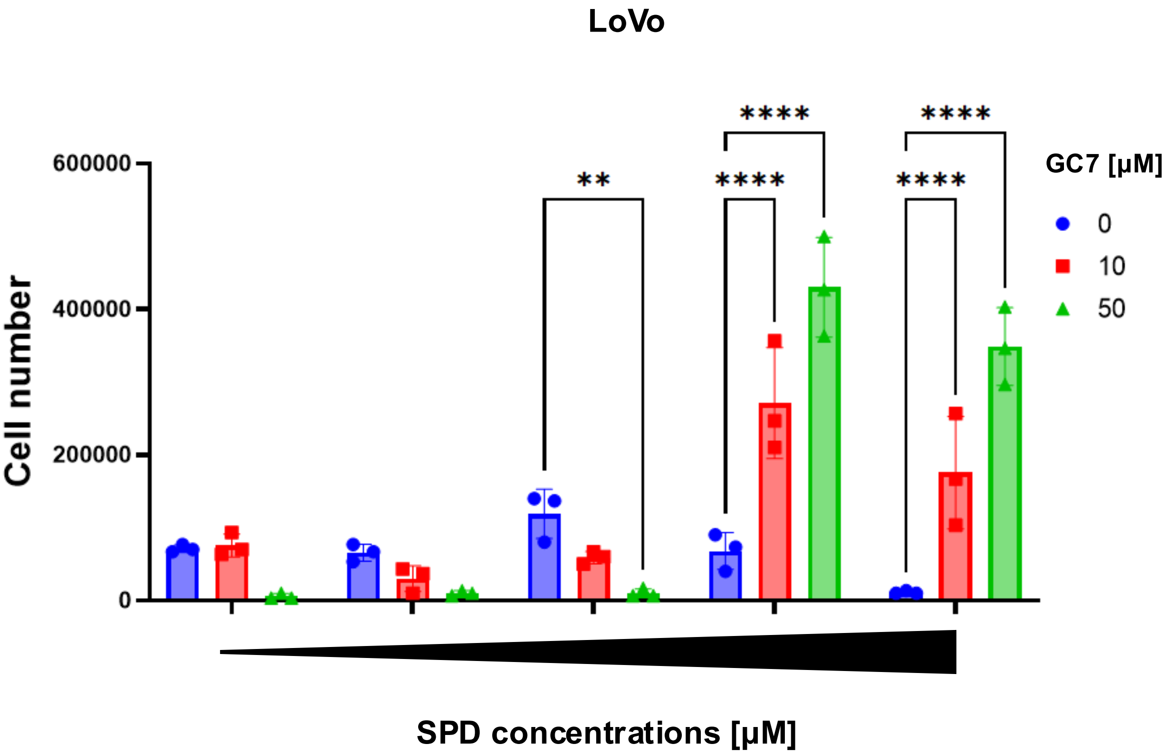

Supplemental Fig. 3:
Cell proliferation assay in LoVo cells (n=3) treated with low doses of SPD (0, 0.01 and 0.1 μM) or high doses (50 and 100μM) and GC7 for 72 hours in DMEM with FBS. Data represent the mean ± SD of experiments performed in triplicate. For statistical analysis, ns=not significant (p>0.05), **p < 0.01, ****p < 0.0001 were determined using Dunnett's multiple comparisons test following Two-way ANOVA.

Supplemental Fig. 4. Blockade of polyamine import prevents proliferation enhancement due to low-doses SPD

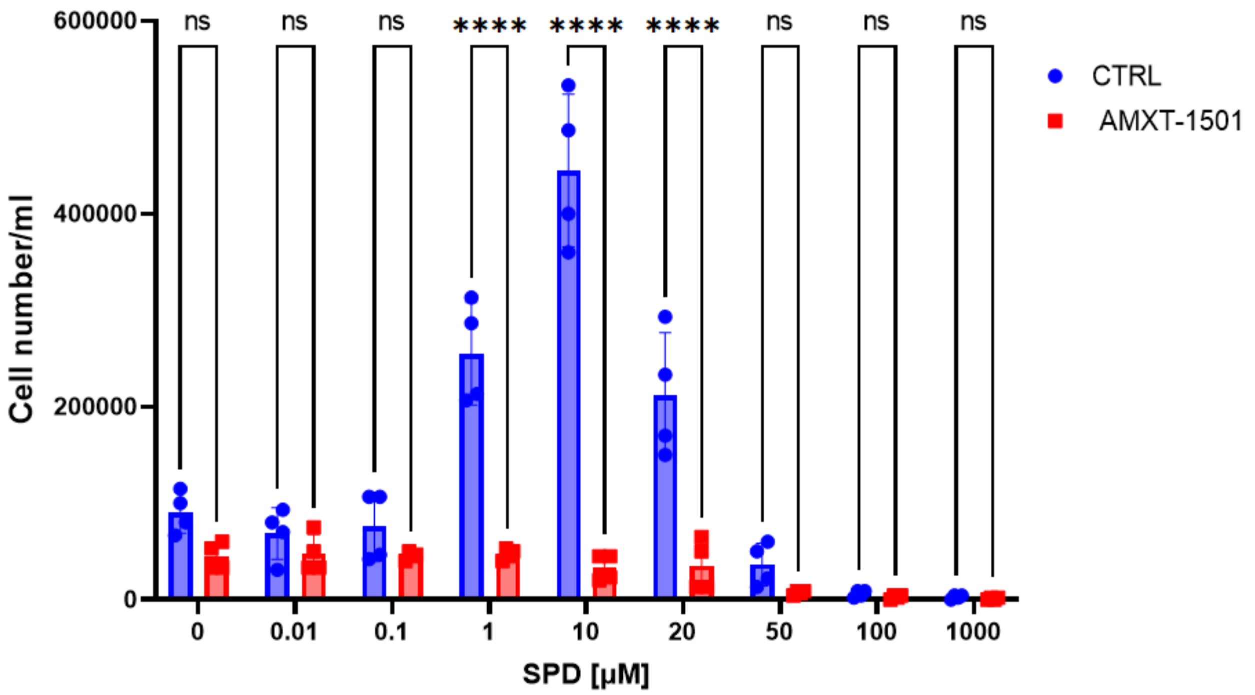

Supplemental Fig. 4:
Cell proliferation assay in HCT116 cells (n=4) treated with the indicated concentrations of SPD and 10µM AMXT-1510 for 72 hours in DMEM with FBS. Data represent the mean ± SD of experiments performed in triplicate. For statistical analysis, ns=not significant ($p>0.05$), ****$p < 0.0001$ were determined using Dunnett's multiple comparisons test following Two-way ANOVA.

Supplemental Table 1. RMSD and docking scores values for the docking assessment from CLO experimental random conformations. Only values for the best performing combination (Plants/PLP) are shown.

Ligand[a]	Conformation[b]	Protein[c] 1TU5B RMSD[d]	1TU5B Score[e]	1TU5A RMSD	1TU5A Score	2PNCA RMSD	2PNCA Score	2PNCB RMSD	2PNCB Score	Best Docked Lock[c]	Best Docked[f] RMSD	Best Docked[f] Score	Average[g] RMSD
2PNCA	EC	15.34	-58.72	15.53	-59.99	2.69	-77.13	2.74	-66.52	2PNCA	2.69	-77.13	1.56
2PNCB		7.86	-59.58	15.12	-59.19	0.42	-77.04	2.48	-65.91	2PNCA	0.42	-77.04	
2PNCA	RC	12.22	-61.60	11.80	-62.69	0.56	-79.83	2.57	-67.62	2PNCA	0.56	-79.83	0.62
2PNCB		13.54	-61.03	11.28	-61.80	0.67	-79.83	1.15	-67.06	2PNCA	0.67	-79.83	

[a]: PDB code from which the CLO ligand was extracted. The last letter indicates the chain.

[b]: EC = experimental conformation; RC = random conformation

[c]: PDB code of the protein used as a receptor, the last letter indicates the chain.

[d]: Root Means Squared Deviation

[e]: PLP score

[f]: RMSD and Score for the best scored docked conformation (low is better)

[g]: Average RMSD value for CLO docking into the 2PNC chains

Supplemental Table 2. Docking score energies of GC7 for active (1TU5) and inactive (2PNC) BSAO conformations.

Key	Lock	Score Energy (kcal/mol)[1]
**GC7**	1TU5	-65,68
**GC7**	2PNC	-67,33

[1]: score obtained by the Plants/PLP docking
